# Acute bilateral blindness due to diffuse outer retinopathy following clear lens exchange: a case report

**DOI:** 10.1186/s12886-023-03171-1

**Published:** 2023-10-23

**Authors:** Nicolas Gurtler, Alice Bughin, Veronika Vaclavik, Eirini Kaisari, Yan Guex-Crosier

**Affiliations:** 1 Jules-Gonin Eye Hospital, FAA, University of Lausanne, Avenue de France 15, 1002 Lausanne, Switzerland; 2 Centre Hospitalier Universitaire Vaudois, Rue du Bugnon 46, 1005 Lausanne, Switzerland

**Keywords:** Autoimmune retinopathy, Outer retinopathy, Toxic, Cataract, Refractive, Case report

## Abstract

**Background:**

As the trend of refractive lens exchange for presbyopia continues to grow, our case report shows the first occurrence of an acute bilateral outer retinopathy following uncomplicated sequential clear lens extraction in an otherwise healthy individual.

**Case presentation:**

A 54-year-old male without significant medical history benefited from a sequential bilateral lens exchange for presbyopia. He then experienced a rapid vision loss in both eyes, accompanied by photopsias and myodesopsias, with symptoms appearing respectively 4 and 3 weeks after the surgeries. Multimodal imaging revealed a fulminant outer retinopathy, leading to a total loss of light perception within a few days. Immediate intravenous corticosteroid therapy was administered, permitting to recover a small area of central visual function in both eyes, enabling shape and color distinction. The primary diagnostic hypothesis is a presumed autoimmune retinopathy, triggered by the cataract extraction, while an alternative diagnosis could be a toxic reaction secondary to the use of intracameral cefuroxime and lidocaine during the surgery.

**Conclusion:**

In this report, the authors describe the first recorded instance of outer retinopathy following cataract surgery. This occurrence raises the possibility of auto-immunization leading to retinal atrophy and vision loss as a potential outcome after undergoing cataract surgery.

**Supplementary Information:**

The online version contains supplementary material available at 10.1186/s12886-023-03171-1.

## Background

Cataract surgery has witnessed an increase in the use of multifocal and extended depth of focus intraocular lenses, leading to a rising trend in refractive lens exchange for presbyopia [1, 2]. Although the overall complication rate remains relatively low, the potential impact can be profound, particularly for the active age group of patients undergoing this surgery [3, 4]. In this report, we present the case of a patient who experienced a fulminant outer retinopathy subsequent to refractive clear lens exchange for presbyopia.

### Case presentation

**Fig. 1:**
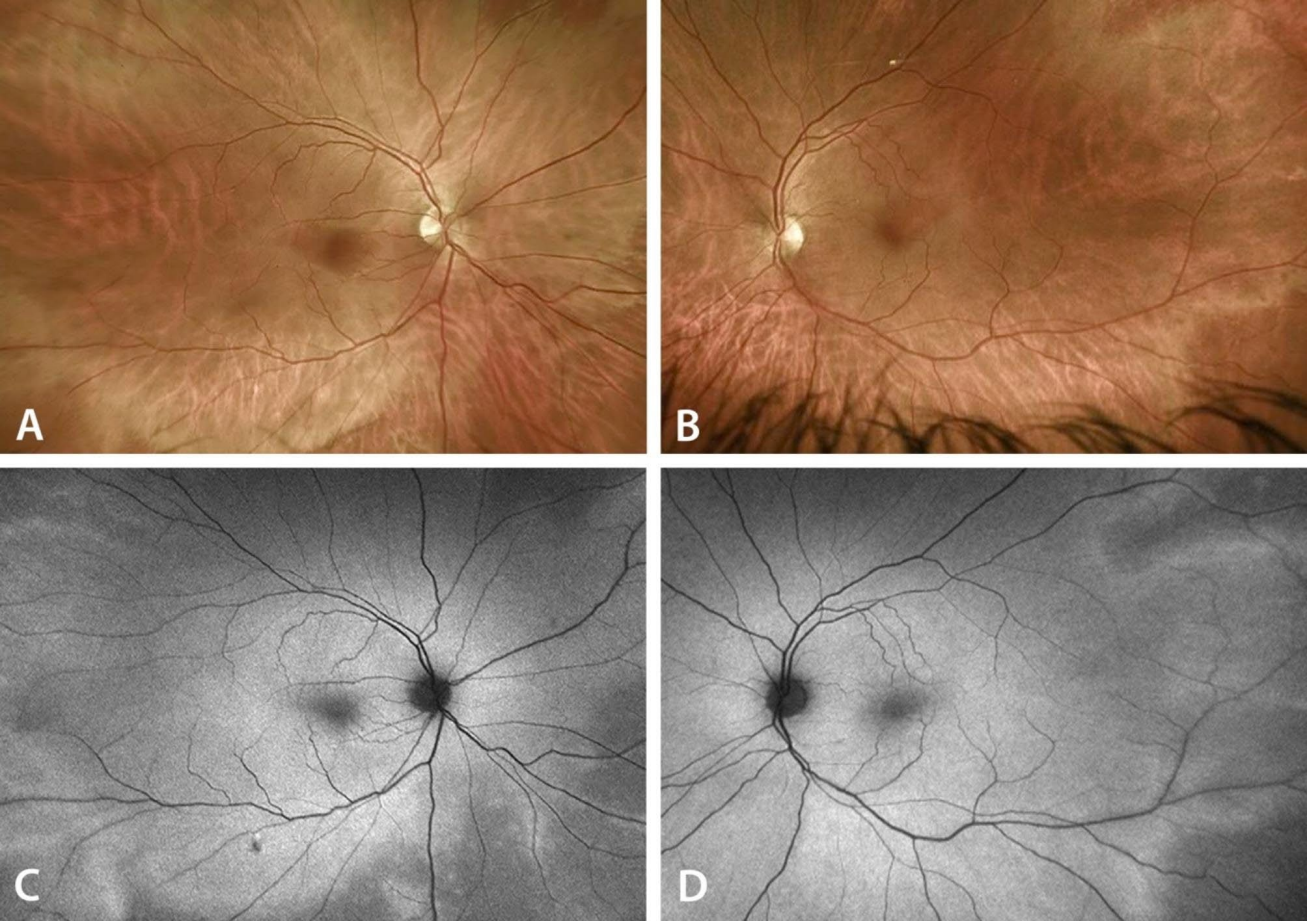
Optos widefield color fundus pictures (**A**,**B**) and autofluorescence frames (**C**,**D**) of the right (**A**,**C**) and left (**B**,**D**) eyes showing retinal perivascular diffuse whitening and hyperautofluorescence

A 54-year-old male with no significant medical history or family history of retinal dystrophies developed rapid bilateral vision loss, accompanied by photopsias and myodesopsias, respectively four and three weeks after refractive cataract surgeries, which were performed nine days apart in the right (Nov. 1st 2022) and left eye (Nov. 10th 2022). The patient reported good visual acuity preoperatively and the post-operative best-corrected visual acuity was 1.0 in both eyes. During the surgeries, intracameral (IC) lidocaine 0.5% and cefuroxime 1 mg/0.1ml (Aprokam®) were administered. Both eyes were implanted with a trifocal intraocular lens (FineVision PodF of 23 diopters). Four days after the onset of symptoms, the patient was examined in our center and presented with a visual acuity of 0.32 in the right eye, hand movements in the left eye, and a tubular visual field bilaterally. The intraocular pressures were 11 mmHg in the right eye and 12 mmHg in the left eye. The anterior segment examination showed an inflammation grading of 1 + of anterior chamber cells, 1 + flare and 0.5 + of anterior vitreous cells in both eyes. The fundoscopy revealed symmetrical, discrete, and diffuse retinal whitening, predominantly located posterior to the equator. This whitening exhibited a perivascular pattern, which was highlighted on the autofluorescence frames. (Fig. 1A-D).

**Fig. 2:**
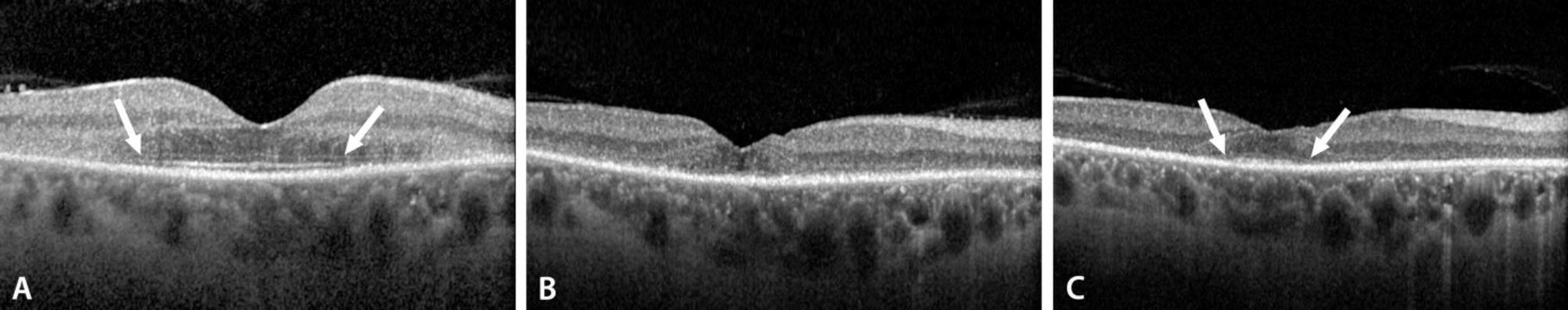
Sequential OCT scans of the right eye demonstrate the rapid disappearance of the perifoveal EZ (white arrows) within 4 days of symptom onset, as well as ASHH lesions (**A**). Two weeks after the onset of symptoms, significant atrophy of the outer nuclear layer is observed (**B**). Following 6 weeks of systemic corticosteroids, there is partial recovery of the foveal EZ (**C**)

The optical coherence tomography (OCT) showed a perifoveal outer retinal disruption, manifesting as an angular sign of Henle fiber layer hyperreflectivity (ASHH), and outer retinal atrophy further in the periphery (Fig. 2A). Both eyes displayed symmetrical outer retinal involvement (Fig. S1 - supplementary figure). The fluorescein/indocyanine green (ICG) angiography showed a mild perfusion delay and no vasculitis. On the first electroretinogram (ERG), conducted 5 days after the onset of symptoms, symmetrical results were observed in both eyes. The rod-specific response was undetectable, the a-wave and b-wave responses were both delayed and of reduced amplitude under scotopic conditions to a standard single flash, 30-Hz flicker and to a single-flash in photopic conditions.

The patient was immediately treated with 4 intravenous infusions of methylprednisolone, twice 250 mg 12 h apart initially, then twice 500 mg/day over 2 consecutive days, after the tests for the main infectious diseases responsible for posterior uveitis yielded negative results (human immunodeficiency virus, syphilis, borreliosis, tuberculosis). Oral prednisone (60 mg/day) was then prescribed, with a gradual tapering over 4 months. Despite the therapy, serial OCTs initially showed rapid disappearance of the foveal ellipsoid zone (EZ), followed by outer retinal atrophy (Fig. 2B), resulting in an absence of light perception in a few days. Following 6 weeks of treatment, the patient experienced a partial recovery of central retinal function, with a visual acuity limited to counting fingers, enabling shape and color perception bilaterally. The OCT scans revealed a partial recuperation of the foveal EZ in both eyes (Fig. 2C). A second ERG was performed 5 months after symptom onset, which showed no detectable responses bilaterally, under both scotopic and photopic conditions.

A neoplasia was ruled out by extensive systemic medical examination (thoracoabdominal computerized tomography (CT) and positron emission tomography (PET) scans) and the serology for anti-recoverin was negative.

### Discussion and conclusions

These findings align with the descriptions of autoimmune retinopathy (AIR) in the literature, which includes cancer-associated retinopathy (CAR), melanoma-associated retinopathy (MAR) and non-paraneoplastic autoimmune retinopathy (npAIR) [5–10]. An extensive medical screening to investigate the possibility of an associated tumor was conducted, but no neoplasia was found. However, vision loss resulting from CAR can precede the detection of an underlying tumor by several years, depending on the specific type of cancer [11]. Therefore, regular screening should be conducted to rule out the presence of a cancerous tumor in the future.

The remarkable aspect of this case is the rapid progression of the outer retinopathy, resulting in a swift vision loss and outer retinal atrophy. The interval between the onset of symptoms and the initial loss of light perception in both eyes was less than one week, which is rarely the case in the literature. In most patients with AIR, the visual loss occurs over weeks to years [9, 10, 12].

Considering the absence of a tumor and the rapid progression, we also raised the possibility of a toxic reaction to the intracameral (IC) cefuroxime or lidocaine used during the cataract surgery. Cefuroxime toxicity is a well-known cause of retinal damage after cataract surgery, [13–16] with various patterns of involvement such as epithelial pigment changes, serous macular detachment, cystoid macular edema, macular infarction, retinal hemorrhages and ellipsoid layer loss [13, 14, 16, 17]. The latter is usually confined to the foveal region and most patients retain useful vision [14]. Most of the retinal toxicity cases secondary to IC cefuroxime result from dosage errors [13, 14, 16]. In our patient’s case a dedicated commercial preparation was used (Aprokam®). Similar cases of toxic posterior syndrome following IC injections were caused by a compounded triamcinolone-moxifloxacin formulation for dropless cataract surgery, which was removed from market due to reports of retinal involvement, secondary to toxic concentrations of poloxamer 407 (binding agent) [18]. Ocular toxicity usually occurs directly after cataract surgery, but may be delayed up to 4 months after surgery in the case of IC vancomycin associated vasculitis [19]. IC lidocain is widely used as an intraocular anesthetic during cataract surgery [20]. It is considered safe, and there has been no reports of definitive retinal toxicity in humans to our knowledge. However, in cases of posterior capsular rupture, the anesthetic can reach the retina and optic nerve, causing transient vision loss that typically recovers in hour to days [21].

Vision loss related to IC lidocaine or cefuroxime usually occurs in the immediate post-operative period [13, 14, 21]. This contrasts with our case where the onset of symptoms was delayed four and three weeks after surgery, and both eyes were simultaneously affected despite the surgeries being performed nine days apart. This atypical presentation suggests an alternative pathophysiological mechanism, possibly an npAIR, triggered by the cataract extraction. Nonetheless, an idiosyncratic reaction cannot be excluded.

According to Nussenblatt, a positive serology indicating the presence of anti-retinal antibodies is a crucial requirement to diagnose AIR [22]. Various antibodies have been associated with this condition, including anti-recoverin, arrestin, α-enolase and transducin-α. Unfortunately, access to the serological testing for this disease is currently limited, and we were only able to test for anti-recoverin, which yielded a negative result. Therefore, we can only state that our patient exhibits clinical features consistent with npAIR but cannot confirm the diagnosis. It is worth noting that seronegative presumed npAIR is frequently reported in the literature [7]. Two case series of patients with presumed npAIR found anti-retinal antibodies in only 41–43% of cases [11, 12]. Approximately 15 target retinal antigens have been identified so far, which only represent a fraction of the potential retinal targets [7, 23]. Regarding the presence of ASHH in the OCT scans: this phenomenon has been recently described in diseases comprising an acute insult to the outer retina, leading to disruption across the entire length of the photoreceptors. ASHH has been documented in multiple cases of CAR and in one case of post-infectious npAIR in a pediatric patient [24–27].

Currently, there is no established treatment protocol for npAIR. Initially a high dose of systemic corticosteroids is usually given, which can be subsequently associated with immunomodulating drugs, intravenous immunoglobulins, plasmapheresis and biologics [7, 28]. Following treatment, improvement in visual acuity and visual field can be expected. In a cohort of patients with npAIR who received various immunosuppressive therapies, 15 of the 24 (62.5%) showed improvement [29]. In our patient, the serial OCT scans revealed a partial recovery of the foveal EZ following 6 weeks of treatment. This phenomenon has been previously observed in a case series of npAIR patients, with 3 out of 10 eyes (23%) displaying similar improvement [6].

Although acute zonular occult outer retinopathy (AZOOR) was initially considered as a potential diagnosis due to similarities with our patient’s case, there were notable differences observed [30, 31]. Our patient experienced bilateral symmetrical loss of visual acuity and a peripheral concentric contraction of the visual field within a week, with foveal involvement leading to an absence of light perception initially. Additionally, discrete anterior chamber and vitreous inflammation were present. In contrast to AZOOR, the fundus examination in our patient revealed a diffuse pale aspect instead of the typical normal appearance or the characteristic whitish retinal ring observed in acute annular outer retinopathy (AAOR) [32, 33].

To our knowledge this is the first case report of diffuse outer retinopathy leading to outer retinal atrophy occurring after sequential uneventful clear lens extraction in an otherwise healthy individual. This occurrence raises the possibility of auto-immunization leading to retinal atrophy and vision loss as a potential outcome after undergoing cataract surgery. Further case reports may shed light on the exact mechanism of this extremely rare complication.

### Electronic supplementary material

Below is the link to the electronic supplementary material.

Supplementary Material 1

## Data Availability

Not applicable.

## Abbreviations

IC: intracameral
OCT: optical coherence tomography
ASHH: angular sign of Henle fiber layer hyperreflectivity
ICG: indocyanine green
ERG: electroretinogram
EZ: ellipsoid zone
CT: computerized tomography
PET: positron emission tomography
AIR: autoimmune retinopathy
CAR: cancer-associated retinopathy
MAR: melanoma-associated retinopathy
npAIR: non-paraneoplastic autoimmune retinopathy
AZOOR: acute zonular occult outer retinopathy
AAOR: acute annular outer retinopathy
