# Supplementary material for: Acute bilateral blindness due to diffuse outer retinopathy following clear lens exchange: a case report
Source: BMC Ophthalmol. 2023 Oct 23;23:428. doi: 10.1186/s12886-023-03171-1 (PMC10594781; doi:10.1186/s12886-023-03171-1)
Supplement: Supplementary file 1 — Supplementary Material 1 [file 12886_2023_3171_MOESM1_ESM.docx]

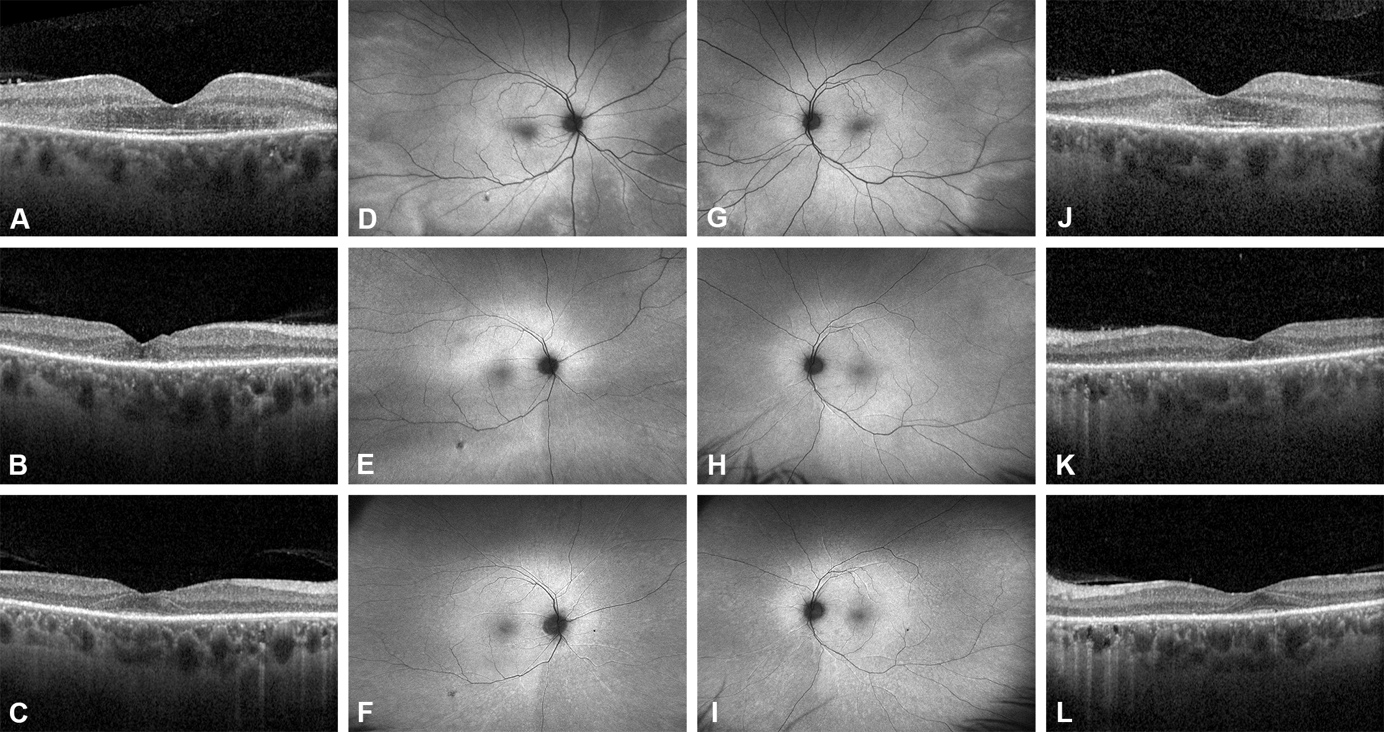


Figure S1: OCT scans (A-C, J-L) and autofluorescence frames (G-I) were obtained for both the right (A-F) and left eye (G-L) at three different time points: 4 days (A, D, G, J), 2 weeks (B, E, H, K), and 6 weeks (C, F, I, L) after the onset of symptoms.

The initial OCT scans displayed a disruption of the outer retina, including ASHH lesions and the disappearance of the peripheral ellipsoid zone (EZ). After 2 weeks, a complete absence of the EZ was observed. At 6 weeks, following immunosuppressive treatment, there was partial remission of the foveal EZ.

The autofluorescence frames revealed a slight decrease in hyperreflectivity, along with the diffuse appearance of minor hyperfluorescent dots at the 6-week mark (F, I).
